# Impact of chlorogenic acid on submandibular salivary gland and liver of albino rats exposed to sodium nitrite

**DOI:** 10.1186/s12903-024-04661-4

**Published:** 2024-08-08

**Authors:** Elham H. Ahmed, Mohammed Abdelrahim Abdeen, Samar Soliman

**Affiliations:** 1 Department of Oral Biology, Faculty of Dentistry, Mansoura University, Mansoura, Egypt; 2 Department of Supplementary Medical Science (Human Anatomy), Faculty of Dentistry, Sinai University, North Sinai, Egypt; 3 Department of Oral Pathology, Faculty of Dentistry, Mansoura University, Mansoura, Egypt

**Keywords:** Sodium nitrite, Chlorogenic acid, Interleukin 6, Malondialdehyde, Superoxide dismutase

## Abstract

**Aim:**

The aim of the present study is to show how sodium nitrite alters the histology of submandibular salivary glands and livers of Albino rats, as well as how chlorogenic acid may have therapeutic benefits.

**Methods:**

A sample size of thirty male Sprague Dawley Albino rats weighing between 100 and 150 g (5-6 weeks old) was randomly allocated into 3 equal groups. Group I: rats were used as controls and were given phosphate buffer solution, whereas Group II: rats were given an 80 mg/kg sodium nitrites (SN) daily dissolved in distilled water. The rats in Group III were given a daily dose of 80 mg/kg SN dissolved in distilled water and after 6 hours each rat received 50 mg/mL freshly prepared chlorogenic acid (CGA) every other day. For 12 weeks, all treatment modalities will be administered orally, every day. After the experiment, all rats were euthanized. Samples from salivary glands and livers were processed and stained with H&E and interleukin 6 (IL 6). Malondialdehyde (MDA) and superoxide dismutase (SOD) enzymes were detected using an ELISA assay.

**Results:**

Groups III had nearly comparable findings to Group I regarding histological pattern with normal submandibular glands and livers features. Group III salivary gland treated with CGA exhibited higher SOD levels (20.60±4.81 U/g) in comparison to the SN group, and lower MDA levels (111.58±28.28 nmol/mg) in comparison to the SN treated samples. In comparison to the SN group, CGA treatment significantly reduced MDA levels in liver samples (167.56±21.17 nmol/mg) and raised SOD (30.85±6.77 U/g).

**Conclusions:**

Chlorogenic acid has a protective effect against salivary gland and liver toxicity induced by SN in rats. This was mediated via the anti-inflammatory and antioxidative properties of CGA and the restoration of oxidant/antioxidant balance in rat salivary gland and liver.

## Background

Humans are constantly subjected to various chemicals, including food additives. There is a growing awareness that many of these chemicals may be harmful to human health. Sodium nitrite (NaNO2) (SN) is considered one of the most important food additives [1]. In the food industry, SN is frequently used for color fixation and fish and meat products preservation. It inhibits lipid oxidation and improves flavor and postpones rancidity. Additionally, it prevents the growth of microorganisms [2]. It has been reported that low physiological concentrations (0.45-23 μM) of nitrite can cause several potential health benefits [3]. However, long-term exposure to even low doses of nitrite at high concentrations can have detrimental effects on health and, in rare cases, even result in death [2]. Adverse health effects, such as birth abnormalities, respiratory tract illnesses, nervous system damage, and paralysis, are brought on by long-term exposure to low levels of nitrite [4]. Long-term nitrite exposure can also result in mutagenicity and carcinogenicity [5, 6]. Oxidative damage is one of the main mechanisms by which nitrite exerts its toxicity. Numerous in vivo and in vitro investigations have demonstrated nitrite toxicity, which occurs by oxidative stress and includes hepatotoxicity, nephrotoxicity, deregulation of inflammatory responses, and tissue injury [7–10]. This is supported by reports that antioxidants can improve nitrite toxicity [11, 12]. Also, degenerative changes in the organs of nitrite-treated mice have been reported [2]. Furthermore, SN toxicity can affect salivary glands inducing inflammatory response and xerostomia affecting the general wellbeing [13]. Research must be focused on understanding the molecular processes of nitrite-induced toxicity since nitrate/nitrite-contaminated areas are seeing an increase in the occurrences of health problems in humans and animals [14], additionally, interest in natural and herbal substances has been growing daily. to be used as an alternative for treatment and prevention of several chronic diseases. Due to their numerous potential beneficial effects relating to their antioxidant and anti-inflammatory qualities, phenolic acids have recently attracted a lot of interest [15]. One of the most readily available phenolic acid components in food, chlorogenic acid (CGA) is derived mostly from coffee and various fruit types. It is a strong antioxidant that can lower intracellular redox states that are out of equilibrium [15, 16]. Additionally, in vitro research has that CGA primarily inhibits inflammation by scavenging reactive nitrogen and oxygen species. Additionally, CGA enhances antitumor immunity by blocking the NF-κB/EMT signaling pathway, which has anticancer and anti-metastatic effects. This suggests that CGA could be a viable option for cancer therapy [17]. Intoxicated salivary glands could directly affect general wellbeing due to xerostomia and decreased immunity of oral cavity [18]. The liver is susceptible to injuries by chemical compounds and xenobiotic because it is often the site of metabolism, and it is where some chemicals concentrate and become bio-activated [19]. There is a link between liver and salivary gland tumors; liver metastasis from salivary gland cancer [20] and metastasis of hepatocellular carcinoma to salivary glands [21] was reported. Owing to the mechanism of SN toxicity and the characters of CGA in reducing the inflammation and inducing antioxidant defense, the present work was performed to investigate the possible effect of CGA in reducing the toxic effect of SN in submandibular salivary glands and livers.

## Methods

### Animal subjects and sample size

The sample size was determined using G*Power 3.1.9.2 software (Heinrich-Heine-Universitat Dusseldorf, Germany). An a priori analysis employing ANOVA (fixed-effects, special, main effects, and interactions) was carried out to compute the required sample size. The input parameters were effect size (f = 0.40), power (power = 0.95), numerator (df = 4) and α error probability (0.05). The predictor variables were three groups and one examination period. Three groups of thirty male Sprague Dawley albino rats each weighing between 100 and 150 grams (5-6 weeks old), were randomly assigned. The protocol that was adhered to for all experiment procedures was approved by the Ethics Committee of the Faculty of Dentistry at Mansoura University in Egypt code number MU-ACUC (DENT.R.23.01.5). The rats were kept in separate cages with tap water and standard rodent diet (There was a free access to food and water). Additionally, a 12:12 light-dark cycle, a 22 ºC temperature fluctuation, and 65–70% relative humidity was all part of their light-controlled room. This experiment followed the ARRIVE Checklist (https://www.nc3rs.org.uk/arrive-guidelines) and Animal Research: Reporting in vivo research protocols. All rats were randomly and equally divided into three groups. (1) Control group received phosphate buffer solution. (2) Sodium nitrite-treated (SN) group, rats were treated daily with (80 mg/kg) SN dissolved in distilled water (Sigma Aldrich, St Louis, MO, USA) (at 10:00 am) [22]. (3) Chlorogenic acid treated group, the rats were treated daily with (80 mg/kg) SN diluted in distilled water at (10:00 am) and received CGA (50 mg/mL) (at 4:00 pm) (Sigma Aldrich, St. Louis, MO, USA) that was prepared freshly every other day [23]. All treatment modalities will be given orally and daily for 12 weeks. Body weights were measured weekly. Each rat's weight gain or loss (measured in grams) was determined by deducting its body weight on the day of scarification from its initial weight at the start of the experiment. All rats were anesthetized, and then then euthanized by overdose of halothane [24]. Samples from the liver and submandibular salivary glands were taken and processed for further analysis.

### Determination of levels of malondialdehyde (MDA) and superoxide dismutase (SOD) in submandibular salivary glands (SMG) and liver tissues using ELISA test

Malondialdehyde (nmol/mg) and superoxide dismutase (U/g) were measured using an ELISA as indicators of lipid peroxidation. The right halves of the SMG and liver were washed in accordance with the manufacturer's instructions, removing any extra blood with ice cold phosphate-buffered saline. Butylated hydroxytoluene and a proteolysis inhibitor were added to the samples to prevent oxidation and proteolysis. After homogenization, the samples were stored in 20 mL of 1x phosphate buffer saline for the night at < -20 °C. The homogenate was centrifuged for 5 minutes at 5,000 x g to break the cell membranes and remove any particle materials after two freeze-thaw cycles. The supernatant was then separated and stored at < –20 °C. The rat malondialdehyde and catalase ELISA test kits were supplied by My BioSource, and a Ray to RT 2100C microplate reader was utilized. The acquired samples were stored at -80 °C and then allowed to come to room temperature before being used. As directed by the manufacturer of each kit, tissue samples were added to the experimental wells of the microplate, with a volume ranging from 50 to 100 μl per well. Subsequently, 50–100 μl of the antibody mix per well were added to the experimental and control wells. Before being shaken on a microplate shaker, the microplates were sealed with an adhesive and let to stand at room temperature for two hours. Three gentle washes using 350 μl of wash buffer per well were performed on the experimental wells. Following the addition of 200 μl/well of the antibody conjugate, the plate was shaken for an hour at room temperature in the dark. After that, 200 µl of the substrate solution was added to each well, and the shaker plate was allowed to incubate for 30 minutes. Upon adding 50 microliters of stop solution to each well, the wells' color changed to yellow. The absorbance was measured at 450 nm within 30 minutes of applying the stop solution (results at 570 nm subtracted) Tables 1, 2 and 3.

**Table 1:** Descriptive statistics for the values of Alkaline phosphatase (ALP) (IU/L) and bilirubin (T. BiL) (mg/dL) levels in the blood after 1&2&3 moths(M) of the studied groups

	**Mean ± SD**			**Tukey's HSD Post-hoc test**			
	**Control group**	**SN group**	**CGA group**	***P* value**	***P1*** **Control vs. SN group**	***P2*** **Control vs. CGA group**	***P3*** **SN vs. CGA groups**
**ALP 1M**	225.33±7.00	304.17±22.95	248.83±5.26	≤0.001*	≤0.001*	≤0.001*	≤0.001*
**ALP 2M**	225.67±6.97	350.17±13.68	255.50±5.20	≤0.001*	≤0.001*	≤0.001*	≤0.001*
**ALP 3M**	225.33±7.00	404.67±22.33	280.50±42.15	≤0.001*	≤0.001*	0.010*	≤0.001*
**T. BiL 1M**	0.18±0.05	0.43±0.05	0.36±0.05	≤0.001*	≤0.001*	≤0.001*	**0.057**
**T. BiL 2M**	0.20±0.02	0.63±0.09	0.41±0.05	≤0.001*	≤0.001*	≤0.001*	≤0.001*
**T. BiL 3M**	0.19±0.05	0.95±0.09	0.50±0.03	≤0.001*	≤0.001*	≤0.001*	≤0.001*

**Table 2:** Descriptive statistics for the values of MDA and SOD levels in liver and submandibular gland of the studied groups

Mean ± SD					**Tukey's HSD Post-hoc test**			
		**Control group**	**SN group**	**CGA group**	***P* value**	***P1* Control vs. SN group**	***P2* Control vs. CGA group**	***P3*** **SN vs. CGA groups**
Submandibular gland	**MDA (nmol/g. tissue)**	106.98±19.12	271.98±16.82	111.58±28.28	≤0.001*	≤0.001*	0.005*	0.002*
	**SOD (U/g. tissue)**	39.01±9.74	12.86±1.99	20.60±4.81	≤0.001*	≤0.001*	0.001*	≤0.001*
Liver	**MDA (nmol/g. tissue)**	140.03±27.78	339.70±23.87	167.56±21.17	≤0.001*	≤0.001*	0.003*	0.002*
	**SOD (U/g. tissue)**	54.33±12.43	18.86±3.43	30.85±6.77	≤0.001*	≤0.001*	≤0.001*	≤0.001*

**Table 3:** Descriptive statistics for the values of IL 6 expression in submandibular glad and liver of the studied groups

	**Mean ± SD**			**Tukey's HSD Post-hoc test**			
% area IL 6 expression	**Control group**	**SN group**	**CGA group**	***P* value**	***P1*** **Control vs. SN group**	***P2*** **Control vs. CGA group**	***P3*** **SN vs. CGA group**
Submandibular gland	0.28±0.06	5.35±0.89	0.72±0.27	≤0.001*	≤0.001*	0.004*	≤0.001*
liver	0.30±0.09	8.53±0.83	0.56±0.22	≤0.001*	≤0.001*	0.027*	≤0.001*

^†^ANOVA test

* Tukey's HSD Post-hoc test; results considered significant when the probability of error was less than 5%

### Measuring liver function

Monthly, two ml of blood drained from tail vein of each rat and placed in tubes. These blood samples utilized for detection of serum levels of alkaline phosphatase as well as bilirubin using standard methodologies using commercially available kits.

### Histological and immune-histochemical evaluation

The left halves of the salivary gland and liver specimens were fixed immediately in 10% formaldehyde for a full day after they were prepared in phosphate buffer saline. An automated tissue processor was used for tissue processing. After that, the tissues were imbedded in paraffin blocks and infused with melted paraffin. Serial sections, each 4 µm thick, were removed from each paraffin block, put on covered slides, and left to dry. Histological staining was performed with hematoxylin and eosin (H&E) was used as a routine one to record the changes in the histological architecture of the salivary glands and livers. Rabbit monoclonal IL 6 antibodies were used for immune-histochemical labeling. IL 6 is a key regulator of inflammation and immunological activation. To facilitate antigen retrieval for immunostaining, the parts were deparaffinized, blocked for 30 minutes at 20C using 5% serum, and then heated for 45 minutes at 20C in a citrate buffer.

### Computer-assisted digital image analysis

Slides were photographed using an Olympus® digital camera with a 40 x objective and a 1/2 x photo converter. The photos were analyzed on an Intel® Core I5® based computer using Video Test Morphology® software (Russia), which has a unique built-in mechanism for area and % area measurements and item counting.

### Statistical analysis

Utilizing the Statistical Package of Social Science (SPSS) application for Windows (Standard version 26), all collected data were coded, tabulated, and analyzed. Using the one-sample Kolmogorov-Smirnov test, the data's normality was initially assessed. For data that was regularly distributed, continuous variables were shown as mean ± SD (standard deviation). When comparing more than two groups, the ANOVA test was employed, and the post hoc LSD test was utilized to evaluate comparisons between groups. The 5% level is the set threshold of significance (*p*-value). When the *p* ≤0.05, the results were considered significant. The results are more significant when the *p*-value is less.

## Results

### Changes in body weight

**Fig. 1:**
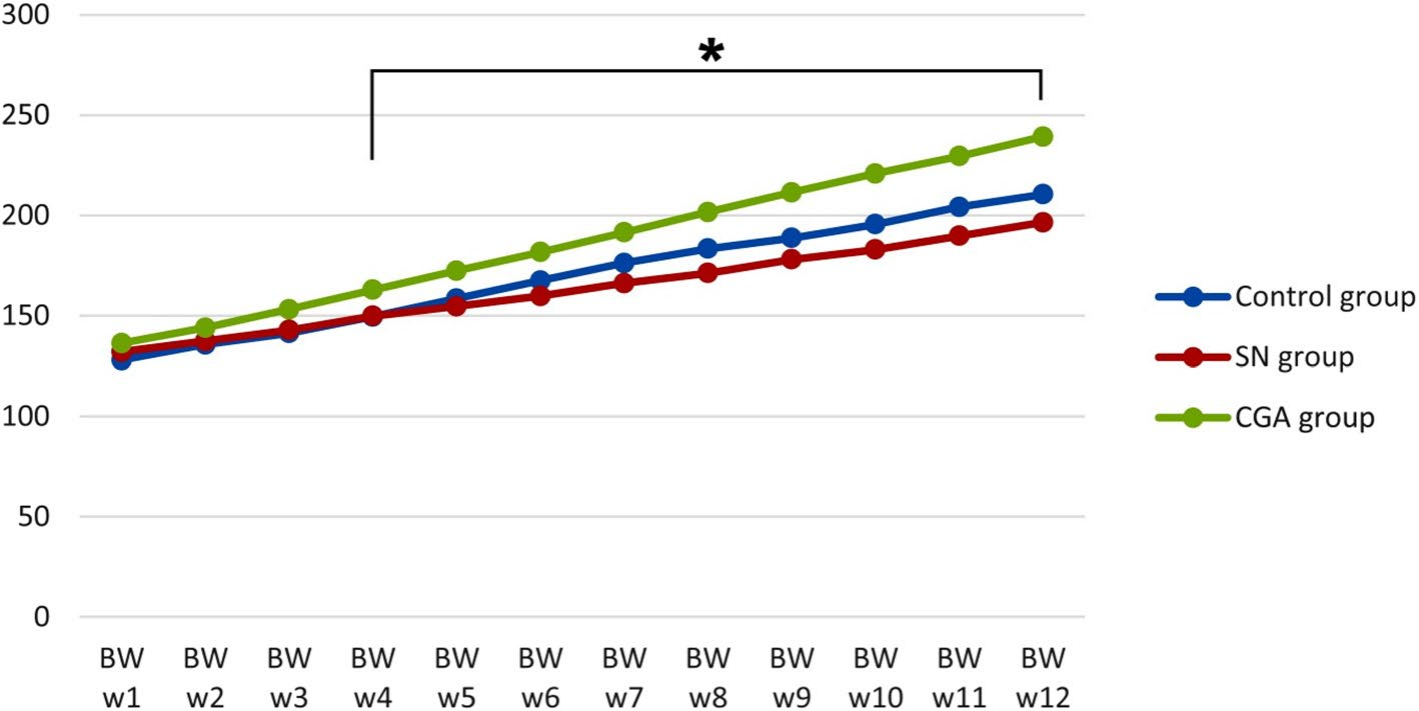
Body weight (BW) gain in grams of the studied groups from week 1(w1) to week 12(w12)

The descriptive statistics showed that rats gained weight in all groups from week 1 to week 12. Meanwhile, rats in control group had weight gain (128.00±10.00*g* and 210.50±16.57*g*) in week 1 and week 12 respectively. Rats in sodium nitrite group had weight gain (132.00±8.55* g* and 196.50±11.43* g*) in week 1 and week 12 respectively. Also rats in chlorogenic acid treated group had weight gain (136.33±9.37 *g* and 239.17±7.25* g*) in week 1 and week 12 respectively. In weeks 1, 2, and 3, an ANOVA test showed that there was an overall non-significant difference in the body weights of all the groups with *p* value (0.329, 0.292 and0.070) respectively but in weeks 4 through 12, there was a significant difference between all groups with *p* value (.007*, 023*, 0.002*, 0.001*≤0.001*, ≤0.001*, ≤0.001*, ≤0.001* and ≤0.001*) respectively (Fig. 1).

### Alkaline phosphatase and bilirubin levels in the blood

**Fig. 2:**
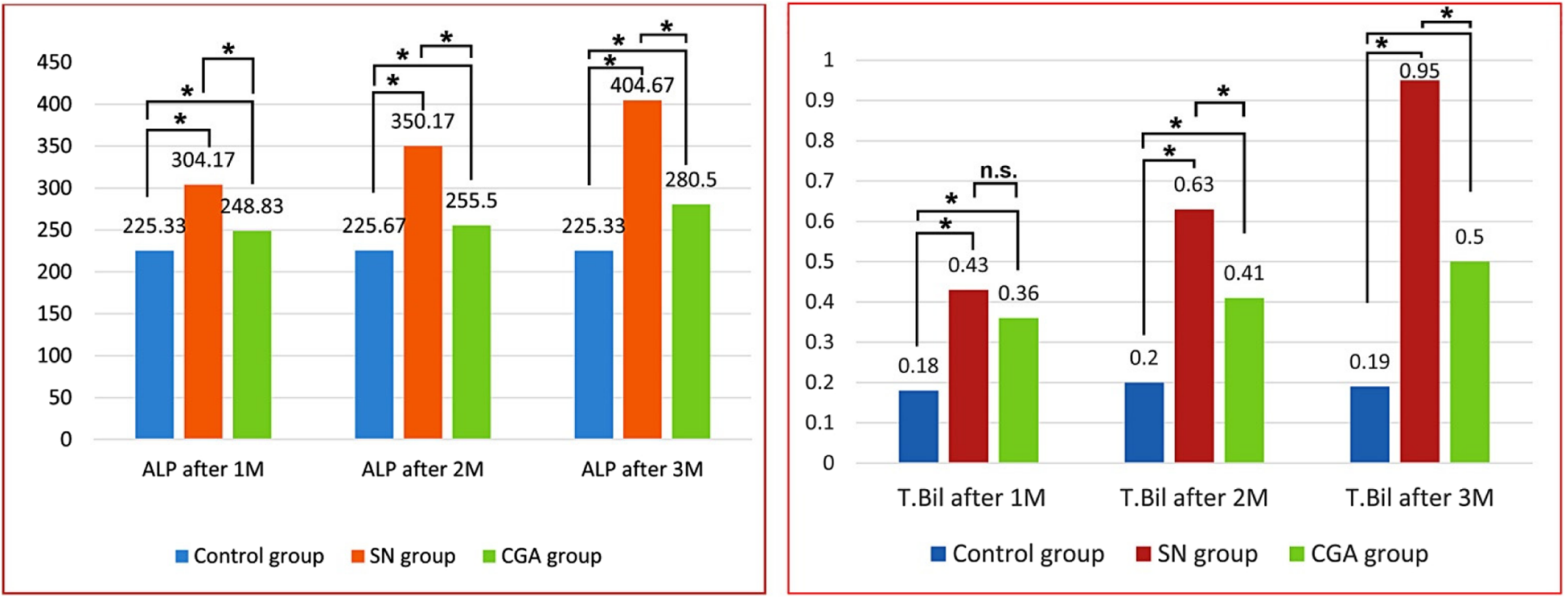
ALP and T. BiL levels in the blood after 1&2&3 moths (M) of the studied groups

An examination of descriptive data showed that the SN group had the highest alkaline phosphatase (304.17±22.95 IU/L. 350.17±13.68 IU/L and 404.67±22.33 IU/L) and bilirubin levels (0.43±0.05 mg/dL, 0.63±0.09 mg/dL and 0.95±0.09 mg/dL) after one, two and three months respectively, while rats in control group had the lowest alkaline phosphatase (225.33±7.00 IU/L. 225.67±6.97 IU/L and 225.33±7.00 IU/L) and bilirubin levels (0.18±0.05 mg/dL, 0.20±0.02 mg/dL. and 19±0.05 mg/dL) after one, two and three months respectively. Statistically, an ANOVA test showed that there was a significant overall difference in each group's alkaline phosphatase and bilirubin levels in the blood after one, two and three months with *p* value ≤0.001 (Fig. 2).

### Malondialdehyde (MDA) and superoxide dismutase expression (SOD) by an ELISA

**Fig. 3:**
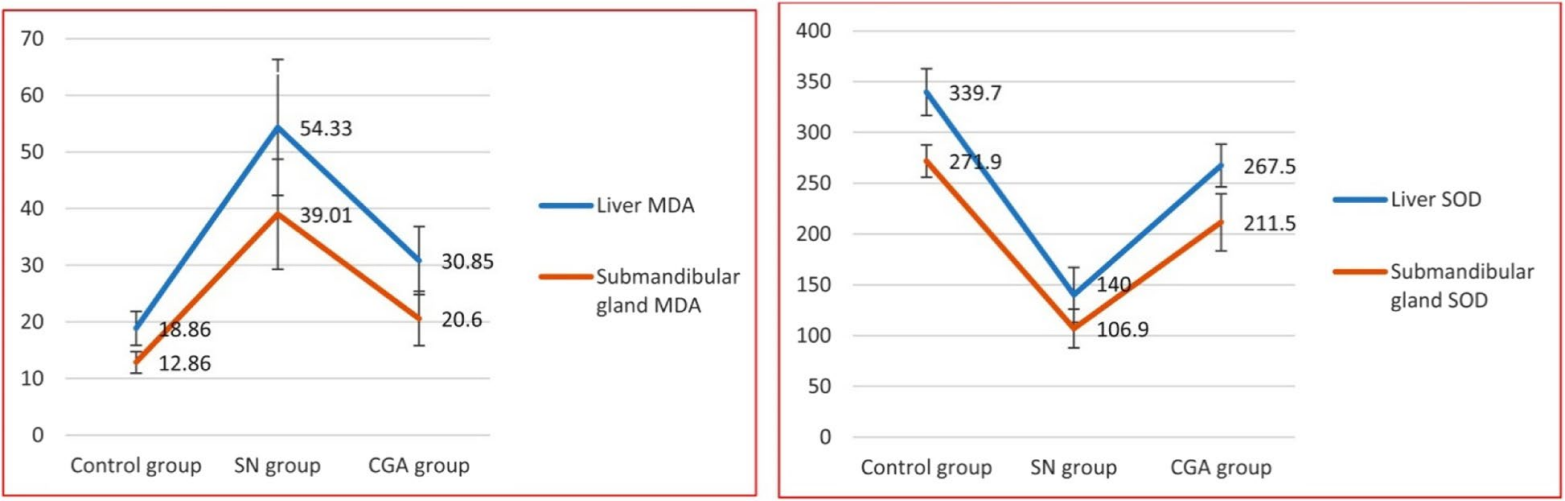
MDA and SOD levels in liver and submandibular gland of the studied groups

In comparison to the control group, salivary gland-intoxicated SN samples exhibited considerably higher MDA levels (271.98±16.82 nmol/mg) and lower SOD (12.86±1.99 U/g). The samples treated with CGA exhibited higher SOD levels (20.60±4.81 U/g) in comparison to the SN group, and lower MDA levels (111.58±28.28 nmol/mg) in comparison to the SN treated samples. In comparison to the control group, the hepatic homogenates from the SN group had a significantly higher MDA level (339.70±23.87 nmol/mg) and a significantly lower SOD (18.86±3.43 U/g). In comparison to the SN group, CGA treatment significantly reduced MDA levels (167.56±21.17 nmol/mg) and raised SOD (30.85±6.77 U/g). Statistically, an ANOVA test showed that there was overall significant difference in each group's MDA and SOD levels in both submandibular salivary gland and liver with *p* value ≤0.001 (Fig. 3).

#### Histopathological changes

**Fig. 4:**
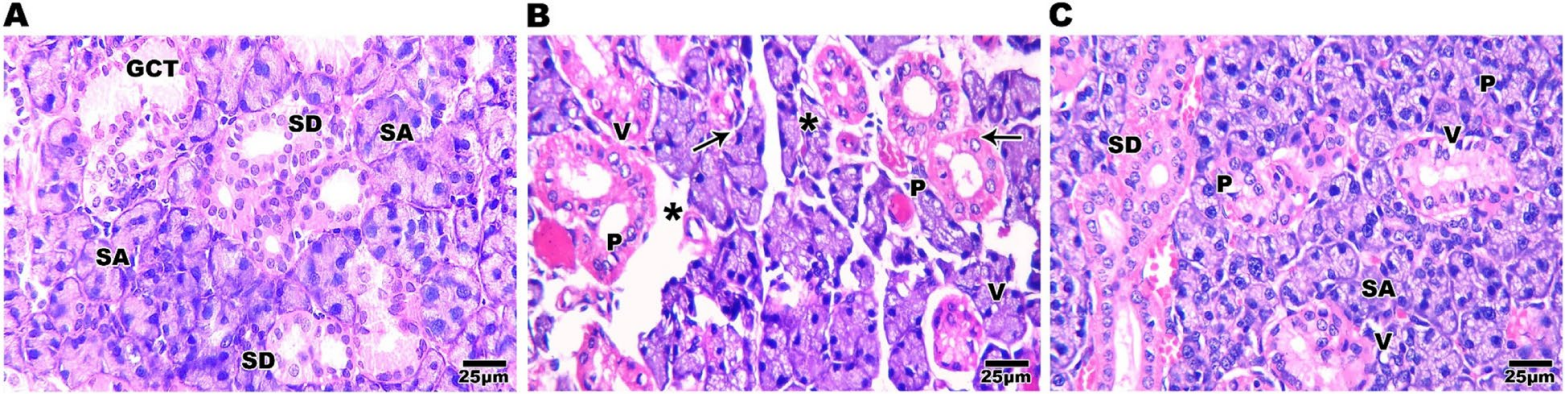
Photomicrograph of salivary gland sections stained with H&E showing normal histological architectural features of rat salivary gland with closely packed serous acini (SA) lined with eosinophilic pyramidal cells and basally situated basophilic round nuclei, normal striated ducts (SD) and granular convoluted tubules (GCT) in control group [A]. SN group section showing loss of classical architecture with the loss of an intact appearance and an increase of interstitial connective tissue spaces between severely degenerated serous acini (star), lining cells with nuclear abnormalities and variant shapes (arrow), loss of continuity of cell membrane of acinar cells with pyknotic nuclei (P), and cytoplasmic macro vacuoles (V) [B]. Salivary glands of CGA treated group have preserved histological and architectural features with closely packed serous acini (SA) and striated ducts (SD) with some nuclear pyknosis and macro vacuoles (V) in the acinar and ductal cells (P) [C]. scale bar 25 µm

**Fig. 5:**
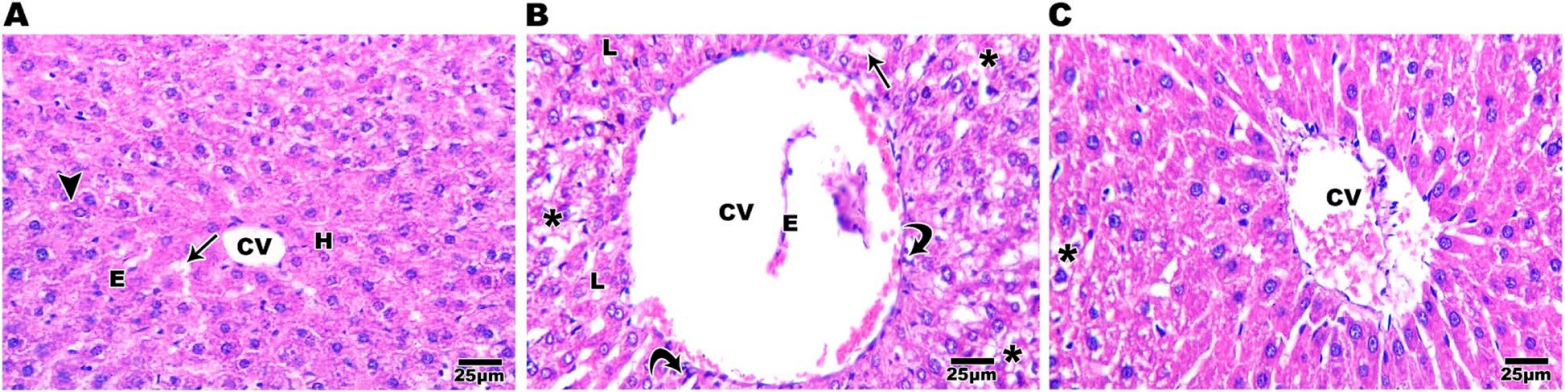
Photomicrograph of liver sections stained with H&E showing hepatocytes cords radiating from central vein and separated by blood sinusoids. H= hepatocyte, arrow= blood sinusoids, CV= central vein, (E)= endothelial cells and arrowhead= binuclear hepatocyte. **A** SN group section showing vacuolar degeneration of hepatocytes (asterisk), lipid droplets (L), disrupted endothelial cell (E), inflammatory cells (curved arrow), dilated congested central vein (cv) and dilated blood sinusoids (arrow) [**B**]. Liver of CGA treated group have improved architecture with reduction in vacuolar degeneration of hepatocytes (asterisk) and congested central vein (cv) [**C**]. scale bar 25 µm

The submandibular salivary gland of the control group had nearly typical rat salivary gland histological architectural features, suggesting proper experimental conditions. Secretory end pieces were predominantly serous acini with basophilic spherical nuclei positioned basally and eosinophilic pyramidal cells lining them. These acini were divided with very fine connective tissue septa, and their lumen was small. Granular convoluted tubules and normal striated ducts were seen (Fig. 4A). In SN group, in submandibular salivary gland, the duct system and acini were both affected by SN toxicity. Moreover, widening and thickening of interstitial spaces, cytoplasmic macro vacuoles at acinar and ductal cells, significant degeneration of acini, and ducts leaving empty spaces in some locations, lining cells with nuclear abnormalities, and loss of the gland's classical architecture with loss of an intact appearance (Fig. 4B). Compared to the SN group, specimens from the submandibular salivary gland group treated with CGA exhibited histological improvements (but were not completely recovered). Maintaining acinar and ductal outlines, as well as many acini and ducts having normal nuclei, were clear indicators of improvement and recovery. Very fine intra-lobular spaces were found. Few cytoplasmic vacuoles were observed at acinar and ductal cells. (Fig. 4C). The liver of control rats shows normal architecture of hepatic lobules with hepatocyte cords radiate from the central vein and divided by endothelium-lined blood sinusoids. Hepatocytes have a big vesicular nucleus and acidophilic cytoplasm; some are binuclear (Fig. 5A). Liver sections from the SN group revealed lower cellular density and vacuolar degeneration of hepatocytes, as well as dilatation and congestion of the central vein with a damaged endothelium lining, a few lipid droplets, a few dispersed inflammatory cells, and dilated hepatic sinusoids (Fig. 5B). When compared to the SN group, the liver specimens from the CGA treated group had histological improvements (but were not completely recovered). There were clear indications of recovery such as improved hepatic architecture with increased cellular density and obvious reduction of vacuolar degeneration of hepatocytes, but the central vein still congested (Fig. 5C).

#### Interleukin 6 immuno-histochemical (IHC) results

**Fig. 6:**
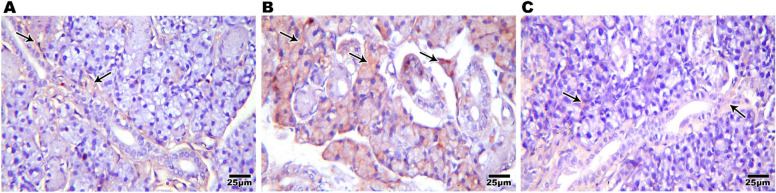
Photomicrograph of rat's salivary gland immune-labeled with anti- IL 6 showing weak immune-reactivity in control group (**A**), a positive reaction that appears with cytoplasmic brown coloration (arrow) within the acinar and ductal cells with the highest level of immuno-reactivity in SN group (**B**) while salivary gland (**C**) of CGA group showed mild immune-reactivity (IHCX400)

**Fig. 7:**
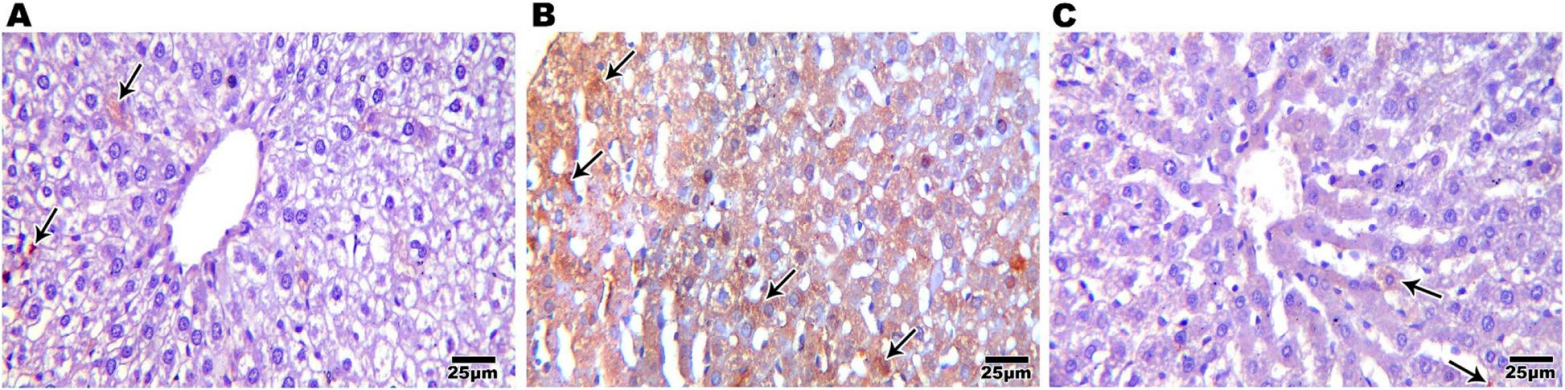
Photomicrograph of rat's liver immune-labeled with anti- IL 6 showing weak immune-reactivity in control group (**A**), a positive reaction that appears with cytoplasmic brown coloration (arrow) with the highest level of immuno-reactivity in SN group (**B**) while liver of CGA group showed mild immune-reactivity (**C**) (IHCX400)

**Fig. 8:**
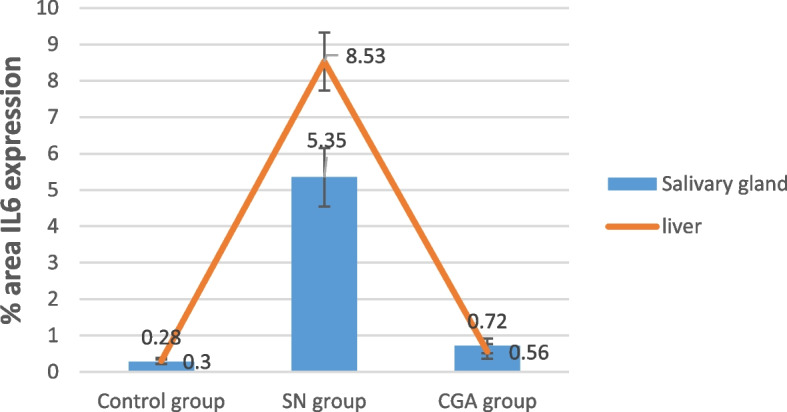
IL 6 expression in submandibular gland and liver of the studied groups

Testing IL6 as a marker of immune system activation showed brown positive IHC staining in submandibular gland (0.28±0.06%) (Fig. 6A) and liver (0.30±0.09%) sections from control group (Fig 7A). Increased brown positive IHC staining in submandibular gland while the CGA treated group revealed fewer brown stained cells (0.72±0.27%) (Fig. 6C), sections from the SN group showed positive reactivity (5.35±0.89%), particularly around the severely vacuolated ducts and morphologically deformed acini (Fig. 6B). Increased brown positive IHC staining (8.53±0.83%) was seen in liver slices from the SN treated group (Fig. 7B). However, hepatic sections from the CGA group showed a significant decrease in brown color (0.56±0.22%) (Fig 7C). An ANOVA test showed that there was overall significant difference between all groups regarding IL6 expression in both submandibular salivary gland and liver with *p* value ≤0.001 (Figure 8)

## Discussion

Sodium nitrite is one of these harmful pollutants that has an impact on human and animal health all over the world [2]. SN is widely used in the food industry for enhancing the taste and the color of meat, preventing the rancidity caused by lipid oxidation and as an antimicrobial agent [25]. But increased the exposure to high levels or even prolonged exposure to low level of SN lead to the formation of harmful compounds such as nitryl chloride, results in cytotoxicity and tissue damage [2, 13]. In the current study, we investigated SN toxicity in submandibular salivary gland and the liver and the effect of CGA as a potent anti-inflammatory and antioxidant. Nitrates and nitrites circulate from the digestive system into the blood, then into saliva, and back into the digestive system [26]. After the active uptake of nitrate from the circulation in the salivary glands and subsequent secretion in saliva, the oral bacteria reduce the nitrate into nitrite. Also, the liver secretes nitrate reductase [27]. Sometimes, nitrite loses an oxygen atom. Then, it turns into nitric oxide (NO), an important molecule that has various functions in the body. It can be toxic in high amounts, but it can also help protect the body [28]. Also, nitrites can be turned into nitrosamine that has harmful effects [29]. On the other hand, Stimulation of a nitrite-NO pathway shows organ-protective effects on oxidative stress and inflammation where the inorganic nitrite target NADPH (Nicotinamide adenine dinucleotide phosphate) oxidase, an important factor to the pathogenesis of various diseases, in the inflammatory vasculature for the antioxidant effect and open a new direction to modulate the inflammatory response [30]. The SN treated group had the highest alkaline phosphatase and bilirubin levels compared to control and CGA treated group that was in accordance with the well documented researches in mammalians that reported the toxic effect of nitrates and nitrites including impairment of reproductive function [31], hepatotoxicity and methaemogobenemia [32], dysregulation of inflammatory responses and tissue injury [33], and endocrine disturbance [34]. Also, Hassan et al. [35] found that the serum of rats given NaNO2 treatment had increased bilirubin levels along with elevated AST, ALT, and ALP enzyme activity. The formation of nitroso-compounds in the stomach's acidic environment can have toxic effects that lead to severe hepatic necrosis, which could explain this [36]. In addition to decreasing blood transaminase and alkaline phosphatase activity as well as total bilirubin levels, Chen Z et al. reported that supplementing with CGA reduced hepatocyte necrosis and inflammatory cell infiltration [37]. We also observed that CGA attenuated these alterations. A 50 mg/kg dose of CGA was also observed to significantly lessen changes in serum levels of α-naphthylisothiocyanate (ANIT), total bile acid, alanine aminotransferase, and alkaline phosphatases caused by this chemical, according to another investigation [38]. They attributed that to the significant protective effect of CGA against lipopolysaccharide -induced liver toxicity and CGA efficiently inhibited the classic toxicity of carbon tetrachloride CCl4-induced liver fibrosis in rats [39, 40]. On the other hand, in the present study SN treated group showed a significantly higher MDA level, and significantly lower SOD compared to CGA group that revealed significantly decreased MDA levels and elevated SOD in hepatic homogenates. Also, Salivary gland intoxicated SN samples showed significantly higher MDA levels and decreased SOD unlike CGA treated samples showed restored levels of SOD and decreased MDA levels compared with SN treated samples. El-Nabarawy et al. observed that as nitrite dosage increased, MDA levels in hepatic and renal oxidative stress significantly increased while antioxidant parameters such reduced glutathione (GSH), SOD, and catalase (CAT) significantly decreased [41]. These findings were in line with those of Abuharfeil et al. [42] who reported that high nitrite doses cause nitrosonium ions, which react with amines and amides to form nitrosamines and nitrosamides, respectively. In vivo, diethyl-nitrosamine causes free radicals to be produced in the liver of rats, and N-nitrosamines can quickly cause oxidative stress. Additionally, as documented by Patsoukis and Georgiou, oxidative cytotoxicity and the negative effects of nitrites in this study generate perturbations in oxidative indicators in rats [43]. Moreover, Ansari et al. [44] observed that superoxide forms and high NO concentrations interact quickly, resulting in lipid peroxidation, tissue degradation, and death. Furthermore, because sodium nitrite depletes the substrates used by SOD and CAT enzymes, it causes a dose-dependent rise in hydrogen peroxide and superoxide radical levels. Lipid peroxidation and the carbonyl content of proteins rise consequently [45]. Regarding the effect of SN on salivary gland, Elsherbini et al. showed that sodium nitrite could increase the MDA level and decrease GSH and total antioxidant capacity (TAC) on the rat’s salivary gland due to the highly reactive property of nitrite, which can reduce, oxidizing or nitrosylating compounds. It can be transformed into other compounds as nitrous acid ad nitric oxide [13]. Regarding CGA, our research was consistent with that of Shi et al., who reported decreased MDA levels and elevated GSH and SOD levels, as well as the involvement of CGA's antioxidant activity in anti-fibrotic effects [46]. Furthermore, Li et al. demonstrated that CGA ameliorated oxidative stress, inflammation, and liver histopathological injury in rats by showing a significant rise in SOD and GSH levels and a decrease in MDA levels following CGA pretreatment [47]. Furthermore, Zha et al. [48] reported that graded levels of dietary CGA supplementation could enhance broiler chickens' growth and antioxidant capacity under normal physiological status and effectively protect against diquat (DQ)-induced oxidative stress by maintaining growth performance and redox status in serum and liver and reducing hepatic inflammatory response. As far as we are aware, no previous research has examined the protective impact of CGA on the salivary glands, where it was found to restore SOD levels and lower MDA levels in comparison to samples treated with SN. Regarding the effect of SN on the histological picture of submandibular salivary gland and liver, our study revealed alterations in granular convoluted ducts and the loss of acinar structures. Furthermore, interstitial gaps were observed to be thickening and broadening in the liver and submandibular salivary gland sections. Liver sections revealed many vacuoles, hepatocyte degeneration, blood sinusoidal dilatation and congestion, and disrupted normal architecture of the hepatic cords. A few papers explained the impact of SN on salivary gland histology. According to Elsherbini et al. [13] in SMG, SN poisoning appeared to have a greater effect on the duct system than the acini. On the other hand, regarding the effect of SN on the liver histology, Ahmadi et al., reported that rats exposed to NaNO2 revealed a reduction in the cell density and a disturbance in the hepatocyte and sinusoidal order and organization. Natural discipline and the hepatic cord's structure were disrupted [49] that agreed with our study. Also, El-Nabarawy et al., [41] observed that as the concentration of NO increased, Significant diffuse vacuolar degeneration was present, along with many pyknotic nuclei and clear hepatic sinusoidal congestion. Additionally, our findings aligned with those of Özen et al. [50] and Abu Aita et al. [51].

In our investigation, the liver and SMG antioxidant defense systems and serum biochemical analysis results were closely correlated with the histological improvements (though incomplete recovery) that the CGA treated group of both SMG and liver demonstrated as compared to the SN group. that was consistent with Hsu et al.'s report that the CGA treatment significantly reduced the significant hepatic injury caused by carbon tetrachloride (CCl4), where there was minor hepatocyte necrosis, inflammatory cell infiltration, and ballooning degeneration [52]. Also, that was the same results in Tian et al., research [53]. Another evidence of damage in both salivary glands and liver by sodium nitrite is the mediation of the inflammation as detected by increased pro-inflammatory cytokine IL 6. In the current research, cytoplasmic immune-reactivity for IL 6 was found in each of the groups under investigation at varying degrees. Both salivary gland and liver of SN group have the highest IL 6 expression than control group. This is in agreement with Soliman et al., 2021 who described that sodium nitrite could promote inflammatory pathways by the increased secretion of cytokines IL 6 [54]. In the same direction, Sun et al. (2006) discovered that exposure to sodium nitrite raised the level of IL 6 in human stomach cells. This could be explained by sodium nitrite-induced increases in oxidative stress and pro-inflammatory cytokine activation [55]. In the current investigation, CGA administration significantly reduced the expression of IL 6 in the liver and salivary gland. According to Buko et al., rats given ethanol showed a reduced inflammatory response involving pro-inflammatory signaling pathways, indicating a strong anti-inflammatory action of CGA [56]. By enhancing antioxidant enzyme activity and reducing lipid peroxidation, CGA repaired the antioxidant defense system. By enhancing the pro-oxidant–antioxidant imbalance, CGA may help avoid damage to the body's organs. The primary pro-inflammatory cytokine that starts an inflammatory cascade involving severe organ function impairments from apoptosis is IL 6 [57]. Therefore, blocking IL 6 production or activity can significantly mitigate the damage to the liver and salivary glands induced by SN poisoning. According to our research, CGA inhibited the pro-inflammatory cytokine IL 6, which had an anti-inflammatory effect and might help prevent damage to the liver and salivary glands.

## Conclusions

We suggest that SN can have serious toxic effects that can damage the salivary glands and liver based on the methodology used and the data found. Rats that get CGA treatment recover more quickly and are better able to fend off SN-induced hepatic and salivary gland damage due to its anti-inflammatory and anti-oxidative properties and the restoration of oxidant/antioxidant balance.

## Recommendation

We recommend to confirm hypo salivation caused by SN and whether CGA had a protective effect by measuring salivary flow. Also ALT/AST tests should be done as specific tests for liver function following the alkaline phosphatase test.

## Data Availability

The data used during the current study are available from the authors on a reasonable request.

## References

[1] Milkowski A, Garg HK, Coughlin JR, Bryan NS. Nutritional epidemiology in the context of nitric oxide biology: a risk-benefit evaluation for dietary nitrite and nitrate. Nitric Oxide. 2010;22(2):110–9. 10.1016/j.niox.2009.08.004. 19748594 10.1016/j.niox.2009.08.004

[2] Ansari FA, Ali SN, Arif H, Khan AA, Mahmood R. Acute oral dose of sodium nitrite induces redox imbalance, DNA damage, metabolic and histological changes in rat intestine. PloS One. 2017;12(4):e0175196. 10.1371/journal.pone.01751963. 28384248 10.1371/journal.pone.01751963 PMC5383256

[3] McNally B, Griffin JL, Roberts LD. Dietary inorganic nitrate: from villain to hero in metabolic disease. Mol Nutr Food Res. 2016;60:67–78. 10.1002/mnfr.201500153. (PMID: 26227946). 26227946 10.1002/mnfr.201500153 PMC4863140

[4] Bryan NS, Loscalzo J, editors. Nitrite and nitrate in human health and disease. Totowa: Humana Press; 2011. Available: http://link.springer.com/ 10.1007/978-1-60761-616-0.

[5] Gui G, Meng S, Li L, Liu B, Liang H, Huangfu C. Sodium nitrite enhanced the potentials of migration and invasion of human hepatocellular carcinoma. SMMC-7721 cells through induction of mitophagy. Yao Xue Xue Bao. 2016; 51: 59–67. PMID: 27405163. 27405163

[6] Zhou L, Zahid M, Anwar MM, Pennington KL, Cohen SM, Wisecarver JL, et al. Suggestive evidence for the induction of colonic aberrant crypts in mice fed sodium nitrite. Nutr Cancer. 2016;68:105–12. 10.1080/01635581.2016.1102298. (PMID: 26699517). 26699517 10.1080/01635581.2016.1102298

[7] Ansari FA, Mahmood R. Sodium nitrite enhances generation of reactive oxygen species that decrease antioxidant power and inhibit plasma membrane redox system of human erythrocytes. Cell Biol Int. 2016;40:887–94. 10.1002/cbin.10628. (PMID: 27214747). 27214747 10.1002/cbin.10628

[8] Jia R, Han C, Lei JL, Liu BL, Huang B, Huo HH, et al. Effects of nitrite exposure on haematological parameters, oxidative stress and apoptosis in juvenile turbot (Scophthalmus maximus). Aquat Toxicol Amst Neth. 2015;169:1–9. 10.1016/j.aquatox.2015.09.016Getrightsandcontent. 10.1016/j.aquatox.2015.09.016Getrightsandcontent 26476021

[9] Kozisek F. Influence of nitrate levels in drinking water on urological malignancies: a community-based cohort study. BJU Int. 2007;99(6):1550–1. 10.1111/j.1464-410X.2007.06970_4.x. 17537226 10.1111/j.1464-410X.2007.06970_4.x

[10] De Saint Blanquat G, Fritsch P, Cazottes C. Effects of dietary nitrite and nitrate on experimentally induced inflammation in the rat. Int J Tissue React. 1983;5(2):173–80 (PMID: 6225743). 6225743

[11] Al-Gayyar MMH, Hassan HM, Alyoussef A, Abbas A, Darweish MM, El-Hawwary AA. Nigella sativa oil attenuates chronic nephrotoxicity induced by oral sodium nitrite: Effects on tissue fibrosis and apoptosis. Redox Rep Commun Free Radic Res. 2016;21:50–60. 10.1179/1351000215Y.0000000035. 10.1179/1351000215Y.0000000035 PMC6837667 26221999

[12] Sherif IO, Al-Gayyar MMH. Antioxidant, anti-inflammatory and hepatoprotective effects of silymarin on hepatic dysfunction induced by sodium nitrite. Eur Cytokine Netw. 2013;24:114–21. 10.1684/ecn.2013.0341. (PMID: 24225033). 24225033 10.1684/ecn.2013.0341

[13] Elsherbini AM, Maysarah NM, El-Sherbiny M, Al-Gayyar MM, Elsherbiny NM. Glycyrrhizic acid ameliorates sodium nitrite-induced lung and salivary gland toxicity: Impact on oxidative stress, inflammation and fibrosis. Hum Exp Toxicol. 2021;40(4):707–21. 10.1177/0960327120964555. (Epub 2020 Oct 8 PMID: 33030083). 33030083 10.1177/0960327120964555

[14] Ansari FA, Khan AA, Mahmood R. Protective effect of carnosine and N-acetylcysteine against sodium nitrite-induced oxidative stress and DNA damage in rat intestine. Environ Sci Pollut Res Int. 2018;25(20):19380–92. 10.1007/s11356-018-2133-9. (Epub 2018 May 4 PMID: 29728968). 29728968 10.1007/s11356-018-2133-9

[15] Naveed M, Hejazi V, Abbas M, Kamboh AA, Khan GJ, Shumzaid M, Ahmad F, Babazadeh D, FangFang X, Modarresi-Ghazani F, WenHua L, XiaoHui Z. Chlorogenic acid (CGA): a pharmacological review and call for further research. Biomed Pharmacother. 2018;97:67–74. 10.1016/j.biopha.2017.10.064. (Epub 2017 Nov 6 PMID: 29080460). 29080460 10.1016/j.biopha.2017.10.064

[16] Tajik N, Tajik M, Mack I, Enck P. The potential effects of chlorogenic acid, the main phenolic components in coffee, on health: a comprehensive review of the literature. Eur J Nutr. 2017;56(7):2215–44. 10.1007/s00394-017-1379-1. 28391515 10.1007/s00394-017-1379-1

[17] Hayakawa S, Ohishi T, Miyoshi N, Oishi Y, Nakamura Y, Isemura M. Anti-cancer effects of green tea epigallocatchin-3-gallate and coffee chlorogenic acid. Molecules. 2020;25(19):4553. 10.3390/molecules25194553. (PMID:33027981;PMCID:PMC7582793). 33027981 10.3390/molecules25194553 PMC7582793

[18] Falk N, Weissferdt A, Kalhor N, Moran CA. Primary pulmonary salivary gland-type tumors: a review and update. Adv Anat Pathol. 2016;23(1):13–23. 10.1097/PAP.0000000000000099. (PMID: 26645458). 26645458 10.1097/PAP.0000000000000099

[19] Karyn Bischoff, Motoko Mukai, Shashi K. Ramaiah, Chapter 15 - Liver Toxicity, Editor(s): Ramesh C. Gupta, Veterinary Toxicology (Third Edition), Academic Press, 2018: 239-257, 10.1016/B978-0-12-811410-0.00015-5.

[20] Zemni I, Tounsi N, Bouraoui I, Slimene M, Sahraoui G, Ayadi MA, Chargui R, Rahal K. A single liver metastasis from adenoid cystic carcinoma of the parotid gland: case report. J Investig Med High Impact Case Rep. 2019;7:2324709619879631. 10.1177/2324709619879631. (PMID: 31556756; PMCID: PMC6764036). 31556756 10.1177/2324709619879631 PMC6764036

[21] Elzouki AN, Elkhider H, Yacout K, Al Muzrakchi A, Al-Thani S, Ismail O. Metastatic hepatocellular carcinoma to parotid glands. Am J Case Rep. 2014;17(15):343–7. 10.12659/AJCR.890661. (PMID:25129420;PMCID:PMC4144943). 10.12659/AJCR.890661 PMC4144943 25129420

[22] Hamdan AM, Al-Gayyar MM, Shams MEE, Alshaman US, Prabahar K, Bagalagel A, Diri R, Noor AO, Almasri D. Thymoquinone therapy remediates elevated brain tissue inflammatory mediators induced by chronic administration of food preservatives. Sci Rep. 2019;9(1):7026. 10.1038/s41598-019-43568-x. 31065039 10.1038/s41598-019-43568-x PMC6505027

[23] Zeng A, Liang X, Zhu S, Liu C, Wang S, Zhang Q, Zhao J, Song L. Chlorogenic acid induces apoptosis, inhibits metastasis, and improves antitumor immunity in breast cancer via the NF-κB signaling pathway. Oncol Rep. 2021;45(2):717–27. 10.3892/or.2020.7891. 33416150 10.3892/or.2020.7891 PMC7757108

[24] Leary SLUW, Anthony R, Cartner S, et al. AVMA guidelines for the euthanasia of animals. Schaumburg, IL: American Veterinary Medical Association, 2013. https://www.example.edu/paper.pdf.

[25] Cvetković D, Živković V, Lukić V, et al. Sodium nitrite food poisoning in one family. Forensic Sci Med Pathol. 2019;15:102–5. 10.1007/s12024-018-0036-1. 30293223 10.1007/s12024-018-0036-1

[26] Lundberg JO, Weitzberg E, Gladwin MT. The nitrate–nitrite–nitric oxide pathway in physiology and therapeutics. Nat Rev Drug Discov. 2008;7(2):156–67. 18167491 10.1038/nrd2466

[27] Eriksson KE, Yang T, Carlström M, Weitzberg E. Organ uptake and release of inorganic nitrate and nitrite in the pig. Nitric Oxide. 2018;75:16–26. 29428840 10.1016/j.niox.2018.02.001

[28] Kröncke KD, Fehsel K, Kolb-Bachofen V. Nitric oxide: cytotoxicity versus cytoprotection—how, why, when, and where? Nitric oxide. 1997;1(2):107–20. 9701050 10.1006/niox.1997.0118

[29] Karwowska M, Kononiuk A. Nitrates/nitrites in food—Risk for nitrosative stress and benefits. Antioxidants. 2020;9(3):241. 32188080 10.3390/antiox9030241 PMC7139399

[30] Sui Y, Tian R, Lu N. NADPH oxidase is a primary target for antioxidant effects by inorganic nitrite in lipopolysaccharide-induced oxidative stress in mice and in macrophage cells. Nitric Oxide. 2019;89:46–53. 31063820 10.1016/j.niox.2019.05.002

[31] Sleight SD, Sinha DP, Uzoukwu M. Effect of sodium nitrate on reproductive performance of pregnant sows. J Am Vet Med Assoc. 1972; 161:819–823. [PubMed] [Google Scholar] [Ref list] .https://pubmed.ncbi.nlm.nih.gov . 4672571

[32] Swann PF. The toxicology of nitrate, nitrite and N-nitroso compounds. J Sci Food Agrec. 1975;26:1761–70. 10.1002/jsfa.2740261119. 10.1002/jsfa.2740261119

[33] Blanquat DG, Fritsch F, Cazotles C. Effect of dietary nitrate and nitrite on experimentally induced inflammation in the rat. Intern J Tiss React. 1983;27:173–80 (PMID: 6225743). 6225743

[34] Jahries G, Hesse VI, Schone LH, Mehnert E. Influence of nitrates and plant goitrgens on thyroid hormone, somated in status and growth of swine. Mj Vet Med. 1986;41:528–30 ([Google Scholar] [Ref list]).

[35] Hassan HA, El-Agmy SM, Gaur RL, Fernando A, Raj MH, Ouhtit A. In vivo evidence of hepato-and reno-protective effect of garlic oil against sodium nitrite-induced oxidative stress. Int J Biol Sci. 2009;5(3):249. 19305642 10.7150/ijbs.5.249 PMC2659008

[36] Kalantari H, Salehi M. The protective effect of garlic oil on hepatotoxicity induced by acetaminophen in mice and comparison with N-acetylcysteine. Saudi Med J. 2001;22:1080–4 ([PubMed] [Google Scholar] [Ref list]). 11802181

[37] Chen Z, Yang Y, Mi S, Fan Q, Sun X, Deng B, et al. Hepatoprotective effect of chlorogenic acid against chronic liver injury in inflammatory rats. J Funct Foods. 2019;62:103540. 10.1016/j.jff.2019.103540

[38] Tan Z, Luo M, Yang J, Cheng Y, Huang J, Lu C, Song D, Ye M, Dai M, Gonzalez FJ, Liu A, Guo B. Chlorogenic acid inhibits cholestatic liver injury induced by α-naphthylisothiocyanate: involvement of STAT3 and NFκB signalling regulation. J Pharm Pharmacol. 2016;68(9):1203–13. 10.1111/jphp.12592. (Epub 2016 Jul 1. PMID: 27367057; PMCID: PMC6300992). 27367057 10.1111/jphp.12592 PMC6300992

[39] Xu Y, et al. Protective effects of chlorogenic acid on acute hepatotoxicity induced by lipopolysaccharide in mice. Inflamm Res. 2010;10:871–7 ([PubMed] [Google Scholar] [Ref list]). 10.1007/s00011-010-0199-z 20405164

[40] Shi H, et al. Chlorogenic acid against carbon tetrachloride-induced liver fibrosis in rats. Eur J Pharmacol. 2009;1–3:119–24 ([PubMed] [Google Scholar] [Ref list]). 10.1016/j.ejphar.2009.09.026 19786014

[41] El-Nabarawy NA, Gouda AS, Khattab MA, Rashed LA. Effects of nitrite graded doses on hepatotoxicity and nephrotoxicity, histopathological alterations, and activation of apoptosis in adult rats. Environ Sci Pollut Res Int. 2020;27(12):14019–32. 10.1007/s11356-020-07901-6. 32036525 10.1007/s11356-020-07901-6

[42] Abuharfeil N, Sarsour E, Hassuneh M. The effect of sodium nitrite on some parameters of the immune system. Food Chem Toxicol. 2001:39(2):119–124. https://pubmed.ncbi.nlm.nih.gov ›. 10.1016/s0278-6915(00)00122-8 11267704

[43] Patsoukis N, Georgiou CD. Effect of sulfite–hydrosulfite and nitrite on thiol redox state, oxidative stress and sclerotial differentiation of filamentous phytopathogenic fungi. Pestic Biochem Physiol. 2007;88:226–35. 10.1016/j.pestbp.2006.11.009. 10.1016/j.pestbp.2006.11.009

[44] Ansari FA, Ali SN, Arif H, Khan AA, Mahmood R. Acute oral dose of sodium nitrite induces redox imbalance, DNA damage, metabolic and histological changes in rat intestine. PLoS One. 2017;12(4):e0175196. 10.1371/journal.pone.0175196. (Borrelli F, ed). 28384248 10.1371/journal.pone.0175196 PMC5383256

[45] Marcus S, Kalaivanam K, Dharmalingam M. Lipid peroxidation in type 2 diabetes mellitus. Int J Diabetes Dev Ctries. 2006;26(1):30. 10.4103/0973-3930.26889. 10.4103/0973-3930.26889

[46] Shi H, Shi A, Dong L, Lu X, Wang Y, Zhao J, Guo X. Chlorogenic acid protects against liver fibrosis in vivo and in vitro through inhibition of oxidative stress. Clin Nutr. 2016;35(6):1366–73. 10.1016/j.clnu.2016.03.002. 27017478 10.1016/j.clnu.2016.03.002

[47] Li K, Feng Z, Wang L, Ma X, Wang L, Liu K, Geng X, Peng C. Chlorogenic acid alleviates hepatic ischemia-reperfusion injury by inhibiting oxidative stress, inflammation, and mitochondria-mediated apoptosis in vivo and in vitro. Inflammation. 2023;46(3):1061–76. 10.1007/s10753-023-01792-8. (PMCID: PMC10188389). 36856879 10.1007/s10753-023-01792-8 PMC10188389

[48] Zha P, Wei L, Liu W, Chen Y, Zhou YJPS. Effects of dietary supplementation with chlorogenic acid on growth performance, antioxidant capacity, and hepatic inflammation in broiler chickens subjected to diquat-induced oxidative stress. 2023;102(3):102479. 10.1016/j.psj.2023.102479. 10.1016/j.psj.2023.102479 PMC9871335 36669355

[49] Ahmadi F, Monfared AL, Shakarami NJAJoP. Protective effect of Zataria multiflora Boiss against sodium nitrite-induced hepatotoxicity in rats. 2022;12(3):213. 10.22038/AJP.2021.18781. 10.22038/AJP.2021.18781 PMC9482715 36186930

[50] Özen H, Kamber U, Karaman M, et al. Histopathologic, biochemical, and genotoxic investigations on chronic sodium nitrite toxicity in mice. Exp Toxicol Pathol. 2014;66(8):367–75. 10.1016/j.etp.2014.05.003. 24947405 10.1016/j.etp.2014.05.003

[51] Abu Aita NA, Mohammed FF. Effect of marjoram oil on the clinicopathological, cytogenetic and histopathological alterations induced by sodium nitrite toxicity in rats. Global Veterinaria. 2014;12(5):606–16. 10.5829/idosi.gv.2014.12.05.83186. 10.5829/idosi.gv.2014.12.05.83186

[52] Hsu YW, Chen YY, Tsai CFJP. Protective effects of chlorogenic acid against carbon tetrachloride-induced hepatotoxicity in mice. 2021;10(1):31. 10.3390/pr10010031.

[53] Tian H, Liu L, Li Z, Liu W, Sun Z, Xu Y, et al. Chinese medicine CGA formula ameliorates liver fibrosis induced by carbon tetrachloride involving inhibition of hepatic apoptosis in rats. 2019; 232:227-35. 10.1016/j.jep.2018.11.027. 10.1016/j.jep.2018.11.027 30471378

[54] Soliman MM, Aldhahrani A, Metwally MMM. Hepatoprotective effect of *Thymus vulgaris* extract on sodium nitrite-induced changes in oxidative stress, antioxidant and inflammatory marker expression. Sci Rep. 2021;11:5747. 10.1038/s41598-021-85264-9. 33707592 10.1038/s41598-021-85264-9 PMC7952422

[55] Sun J, Aoki K, Wang W, Guo A, Misumi J. Sodium nitrite induced cytotoxicity in cultured human gastric epithelial cells. Toxicol In Vitro. 2006;20:1133–8. 10.1016/j.tiv.2006.02.005. 16581224 10.1016/j.tiv.2006.02.005

[56] Buko V, Zavodnik I, Budryn G, Zakłos-Szyda M, Belonovskaya E, Kirko S, Z’ yz’elewicz D, Zakrzeska A, Bakunovich A, Rusin V, et al. Chlorogenic acid protects against advanced alcoholic steatohepatitis in rats viamodulation of redox homeostasis, inflammation, and lipogenesis. Nutrients. 2021;13:4155. 10.3390/nu13114155. 34836410 10.3390/nu13114155 PMC8617701

[57] Xu Y, Chen J, Yu X, Tao W, Jiang F, Yin Z, Liu C. Protective effects of chlorogenic acid on acute hepatotoxicity induced by lipopolysaccharide in mice. Inflamm Res. 2010;59:871–7. 10.1007/s00011-010-0199-z. 20405164 10.1007/s00011-010-0199-z

